# Indoor Air Quality Considerations for Laboratory Animals in Wildfire-Impacted Regions—A Pilot Study

**DOI:** 10.3390/toxics10070387

**Published:** 2022-07-12

**Authors:** Adam Schuller, Ethan S. Walker, Jaclyn M. Goodrich, Matthew Lundgren, Luke Montrose

**Affiliations:** 1 Biomolecular Sciences Graduate Program, Boise State University, 1910 W University Drive, Boise, ID 83725, USA; adamschuller@u.boisestate.edu; 2 Center for Population Health Research, University of Montana, 32 Campus Drive, Missoula, MT 59812, USA; ethan.walker@mso.umt.edu; 3 Department of Environmental Health Sciences, University of Michigan School of Public Health, 1415 Washington Heights, Ann Arbor, MI 48109, USA; gaydojac@umich.edu; 4 Office of Research Compliance, Boise State University, 1910 W University Drive, Boise, ID 83725, USA; mattlundgren@boisestate.edu; 5 Department of Public Health and Population Science, Boise State University, 1910 W University Drive, Boise, ID 83725, USA

**Keywords:** air quality, lab animal, wildfire smoke

## Abstract

**Simple Summary:**

With increasing wildfires in the western US and around the world, it is important to take stock of impacts to humans as well as animals. Fires create smoke, and exposure to wildfire particles is known to negatively impact health. Therefore, we asked if smoke might get into buildings where animal research takes place. Our one-month study provides evidence that smoke does get inside an animal facility and levels can exceed ambient air quality standards that are set to protect public health. More work is needed to establish the impact that indoor smoke exposure might have on research animals, but we suggest these data warrant consideration for air quality monitoring and planning within animal facilities at risk for outdoor smoke events.

**Abstract:**

Wildfire events are increasing across the globe. The smoke generated as a result of this changing fire landscape is potentially more toxic than air pollution from other ambient sources, according to recent studies. This is especially concerning for populations of humans or animals that live downwind of areas that burn frequently, given that ambient exposure to wildfire smoke cannot be easily eliminated. We hypothesized that a significant indoor air pollution risk existed for laboratory animal facilities located proximal to fire-prone areas. Here, we measured real time continuous outdoor and indoor air quality for 28 days at a laboratory animal facility located in the Rocky Mountain region. We demonstrated that during a wildfire event, the indoor air quality of this animal facility is influenced by ambient smoke events. The daily average indoor fine particulate matter value in an animal room exceeded the Environmental Protection Agency’s ambient annual standard 14% of the time and exceeded the World Health Organization’s ambient annual guideline 71% of the time. We further show that specialized cage filtration systems are capable of mitigating air pollution penetrance and could improve an animal’s microenvironment. The potential effects for laboratory animal physiology that occur in response to the exposure levels and durations measured in this study remain to be determined; yet, even acute wildfire exposure events have been previously correlated with significant differences in gene regulatory and metabolic processes in vivo. We believe these findings warrant consideration for indoor laboratory animal facility air quality monitoring and development of smoke exposure prevention and response protocols, especially among facilities located downwind of fire-prone landscapes.

## 1. Introduction

Extreme weather events are significant contributors to adverse health around the globe, and their occurrence has increased substantially with climate change [1]. In particular, wildfires are increasing in size and duration as global temperature increases [2]. This has been associated with a greater health cost burden, mortality, and morbidity world-wide [3,4,5]. The top five years for acres burned in the United States (US) since 1960 have all occurred in the last 15 years and wildfire events in 2020 and 2021 burned more than 15 million combined acres [6]. Consequently, wildfire smoke continues to be a major contributor of particulate matter (PM) in wildfire-prone regions despite downward trends in ambient air pollution elsewhere in the US [7]. Computational models suggest that wildfire smoke currently makes up 25% of total ambient air pollution in the US with estimates that this number could surpass 50% in the next 20 years [8,9].

Wildfire smoke is a complex mixture of chemicals that vary in composition depending on burning conditions and the proximity of the sampling location relative to the source [10]. Despite this, common constituent groups include volatile organic compounds, gaseous pollutants (e.g., carbon monoxide), polycyclic aromatic hydrocarbons, and PM [11]. The smoke from wildfires contains more fine (aerodynamic diameter < 2.5 µm) and ultrafine (aerodynamic diameter < 0.1 µm) PM relative to coarse (2.5 µm < aerodynamic diameter < 10 µm) PM, which is significant because these smaller particles pose greater risks to health given their ability to penetrate deep into the lung and contribute to disease pathology and mortality [12,13,14]. A growing body of data from both animal and human studies suggests that PM_2.5_ from wildfire smoke can impact health more significantly than PM_2.5_ from other sources, due in part to its propensity to produce reactive oxygen species [15,16,17]. Furthermore, the adverse impact of wildfire smoke exposure is not isolated to the pulmonary and cardiovascular systems, but can also affect other systems such as the central nervous system and reproductive organs [18,19].

Wildfire smoke air pollutants can infiltrate structures and impact indoor air quality as well as the health or disease risk of individuals within those structures. This has been observed in schools and commercial buildings [20,21], but has not been studied in other important settings, including laboratory animal facilities. Laboratory animal research is an integral part of many fields including toxicology, pharmacology, and other biomedical sciences. A major benefit of using model organisms in settings such as academia, contract research organizations (CROs), and governmental research institutions is better control of potentially confounding variables. The reproducibility of scientific studies requires the ability to replicate the specific conditions under which the research was carried out. Such conditions would include purposeful as well as inadvertent exposures. Laboratory animals are housed in a wide range of indoor facilities with diverse air quality management systems and are, thus, potentially vulnerable to inhalation exposures, including those that emanate from outside the facility (e.g., wildfire smoke).

Measurement of indoor air quality in animal facilities is not a new concept, but these data are not often collected or reported in the literature as part of the standard facility metrics (e.g., temperature, relative humidity, light/dark cycle, and air change rate) [22]. The sparse data that exist demonstrate that animals in confined spaces are susceptible to air pollution exposure from the macroenvironment (e.g., recirculated building exhaust air) and microenvironment (e.g., dust generated from cage bedding) [23,24,25]. Importantly, the most recent version of the Guide for the Care and Use of Laboratory Animals addresses multiple aspects of air quality, but focuses exclusively on air pollutants generated inside the facility and not on air pollutants that may infiltrate the facility from the ambient environment [26]. This leaves a significant gap in guidance related to monitoring changes in indoor air quality, or exposure-induced health effects, that might subsequently affect experimental data. To help address this gap, our group performed a pilot assessment of indoor air quality using low-cost sensors in a laboratory animal facility located in a wildfire-prone region of the US. Below, we will discuss the collected data, potential impacts on the animal research community, and offer some recommendations.

## 2. Materials and Methods

**Figure 1:**
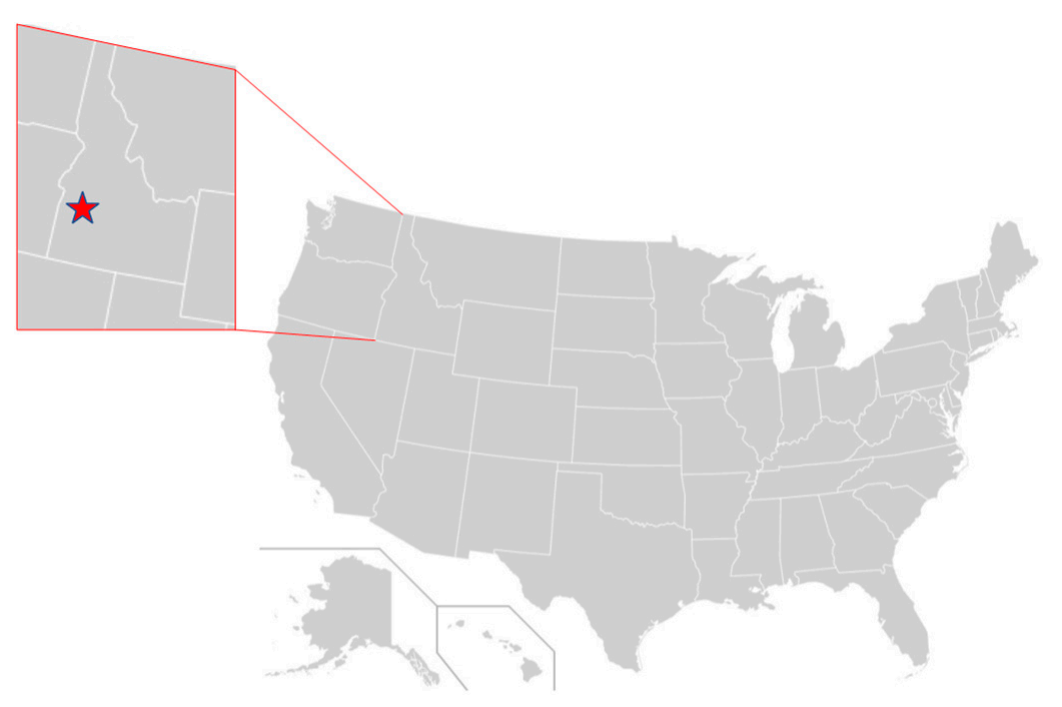
Location of Idaho within the United States and the city of Boise (red star) within the state of Idaho.

*University and facility location*: This pilot study was conducted for a 28-day period from 8 August to 4 September 2021 on the Boise State University campus located in Boise, Idaho (Figure 1). Idaho is a US state situated in the Rocky Mountain region with its capital city of Boise located in the Treasure Valley between the Owyhee and Boise Mountain ranges. The Boise metropolitan area is impacted by smoke generated from wildfires from within Idaho as well as British Columbia, Washington, Oregon and California, depending on weather patterns.

*Facility characteristics:* Air quality sensors were sited at the Boise State University vivarium. This 576 m^2^ facility houses the majority of the animals on campus, which are primarily rodents. The vivarium’s construction in 2015 was funded by a National Institutes of Health (NIH) award and thus was built to the NIH’s stringent Design Requirements Manual (DRM) specifications [27]. The building heating ventilation and air conditioning (HVAC) filters used during the study period were Minimum Efficiency Rating Value (MERV) 15 filter; MERVs are derived from a test method developed by the American Society of Heating, Refrigerating and Air Conditioning Engineers (ASHRAE), and a rating of 15 is given to a filter that removes >85% of particles 0.3–1 µm and >90% of particles 1.0–10 µm [28]. The HVAC system also has a MERV 8 pre-filter for the removal of large dust particles. The air change rate during the study period was greater than 10 per hour.

*Compliance authorization:* The study does not directly involve animals so did not require Institutional Animal Care and Use Committee protocol approval. However, our research team worked closely with the Boise State University Office of Research Compliance at all stages including project development, implementation, and analysis.

**Figure 2:**
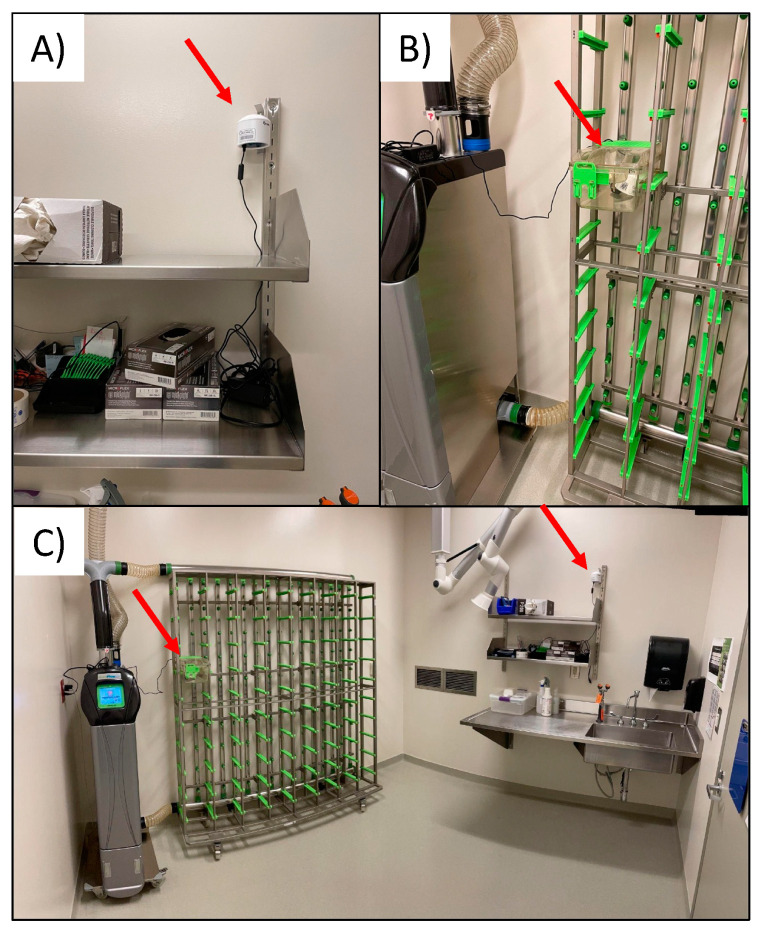
Placement of indoor sensors including (**A**); indoor wall location, (**B**); indoor HEPA cage location, and (**C**); room where both indoor sensors were located.

*Air quality monitoring:* Air quality was monitored indoors and outdoors at the university vivarium. The PurpleAir PA-II (PurpleAir, Inc., Draper, UT, USA) was used and contains two PMS5003 sensors (Plantower, Beijing, China). The PMS5003 estimates particle mass concentrations on the principle of light scatter and these methods are elaborated on further by Sayahi et al. [29]. The PMS5003 reports both mass concentrations (including PM_2.5_) and particle counts at 2-min intervals. Mass concentrations are calculated from particle count data using proprietary algorithms developed by the PMS5003 sensor manufacturer and are provided in two data series which are designated “CF = ATM” and “CF = 1”, respectively [30]. Data from each monitor are transferred via Wi-Fi in real-time to a cloud account and are accessed by the research team. Outdoor data were collected from an established PurpleAir sensor named “Boise State Athletics” which is located on campus and approximately 1km from the animal facility. The indoor location was a negatively pressured animal room which typically houses mice and has no windows and one door. Note that during the course of this air quality study, there were no animals housed in this room. Within the experimental room, two PurpleAir sensors were sited (Figure 2C). The first was located approximately 6 feet above the ground on a wall (Figure 2A). The second was located inside an empty (i.e., no animals or bedding) polycarbonate mouse cage mounted on a Tecniplast (West Chester, Pennsylvania, PA, USA) model GM80 rack with high efficiency particulate air (HEPA) supplied and exhausted air (Figure 2B).

*Analysis:* Analysis was conducted using R version 4.0.4 (The R Foundation for Statistical Computing, Austria). We calculated hourly mean PM_2.5_ concentrations from the raw PurpleAir data collected at 2-min intervals. Prior to calculating hourly mean PM_2.5_, we checked data completeness to ensure that each hour of data collection had at least 15 observations (≥50% of the expected 30 observations per hour at 2-min sampling intervals). Each hour of data had at least 25 observations, so no hourly observations were removed from data analysis. We evaluated agreement between the two identical sensors in each PurpleAir monitor by assessing differences and percent differences for the hourly PM_2.5_ concentrations from the paired sensors within each monitor. Hourly observations (n = 4) were removed from the dataset if the PM_2.5_ concentrations from the paired sensors were different by more than 5 µg/m^3^ and had percent differences larger than two standard deviations [31]. Following this evaluation of sensor agreement, we used the mean hourly CF = 1 PM_2.5_ concentration from paired sensors within each PurpleAir monitor for all subsequent analysis. The mean hourly PM_2.5_ concentrations were corrected using an equation developed by the United States Environmental Protection Agency (EPA) that incorporates PM_2.5_ and humidity data collected by the PurpleAir monitor. Only days with 12+ hours of hourly sampling data were included in statistical analyses. We calculated descriptive statistics for PM_2.5_ concentrations (n, mean, sd, minimum [min], median, maximum [max]) for each PurpleAir monitor across all study days and for wildfire days and non-wildfire days. A suspected wildfire day was classified as a day with mean ambient 24-h PM_2.5_ (as measured by the outdoor PurpleAir monitor) greater than 21 µg/m^3^. A similar cut off to classify a wildfire day has been used previously in multiple studies [32,33]. This definition of a wildfire day assumes that the dominant source of ambient PM_2.5_ during sampling is from wildfire smoke, an assumption supported by an analysis of particulate air pollution in the Northwestern US from 1988 to 2016 [7].

We calculated infiltration efficiency (Finf) using a previously validated recursive modeling approach [34,35,36]. Finf is defined as the fraction of the outdoor PM_2.5_ concentration that penetrates to the indoor environment and remains suspended [34]. It is presented as a unitless number between 0 and 1. We used paired hourly indoor and outdoor PM_2.5_ concentrations from the PurpleAir monitors to calculate Finf. The Finf model is based on the assumption that indoor PM_2.5_ is equal to a fraction of outdoor PM_2.5_ from the current hour, a fraction of indoor PM_2.5_ from the previous hour, and indoor PM_2.5_ from the current hour. Data for the calculation were censored to exclude periods with indoor sources of PM_2.5_ (i.e., periods with a rise in indoor PM_2.5_ without a subsequent rise in outdoor PM_2.5_) [34,35]. Censored data were then used in a linear model with indoor PM_2.5_ (indoor_t_) as the outcome variable, outdoor PM_2.5_ (outdoor_t_) and the previous hour’s indoor PM_2.5_ (indoor_t-1_) as predictor variables, and intercept set to 0:

indoor_t_ = α1(outdoor_t_) + α2(indoor_t-1_) + 0

Model coefficients were then used to calculate F_inf_:

F_inf_ = α1/(1 − α2)

We used this equation to calculate infiltration from the outdoor to indoor sampling locations across all study days and separately for wildfire and non-wildfire days. In addition, we multiplied 24-h outdoor PM _2.5_ concentrations by the estimated Finf to estimate outdoor-generated indoor PM_2.5_ concentrations [37,38]. We divided the outdoor-generated indoor PM_2.5_ concentrations by the total 24-h indoor concentration to estimate the percentage of indoor PM_2.5_ generated from outdoor sources [34]. If the infiltrated concentration was greater than the measured indoor concentration, we set the infiltrated concentration to equal the measured indoor concentration [34].

## 3. Results

For the 28-day study, which took place from 8 August–4 September 2021, corrected daily average concentrations of outdoor, indoor, and HEPA cage PM_2.5_ are shown in Table 1. Outdoor air exhibited a higher daily average PM_2.5_ concentration (26.2 µg/m^3^) than both indoor air (8.9 µg/m^3^) and HEPA cage air (3.1 µg/m^3^) across all study days. This trend persisted even when separating wildfire event days (n = 12) and non-wildfire event days (n = 16). The indoor daily average PM_2.5_ concentration was nearly three times higher on wildfire days compared to non-wildfire days. However, the HEPA cage PM_2.5_ values were not different by wildfire day status and remained consistently low at approximately 3.0 µg/m^3^.

Siting PM_2.5_ sensors both indoors and outdoors at the animal facility allowed for the comparison of these data by three methods including difference, ratio, and Finf, which are shown in Table 2. The average PM_2.5_ outdoor to indoor difference was greater for the HEPA cage (23.1 µg/m^3^) as compared to the room indoor sensor (17.3 µg/m^3^) and this trend was similar for the comparison of the ratio of indoor to outdoor PM_2.5_. For both the room indoor sensor and the HEPA cage, the outdoor to indoor difference was highest on wildfire days (31.1 µg/m^3^ and 42.0 µg/m^3^, respectively). Finf values can range from 0 to 1, with values closer to 0 representing less infiltration of outdoor PM_2.5_ to the indoor environment. Finf for the indoor location was 0.30 (95% Confidence Interval [CI] = 0.21 to 0.43) for all study days, 0.30 (95% CI = 0.17 to 0.50) for wildfire days, and 0.40 (95% CI = 0.29 to 0.54) for non-wildfire days. For the HEPA cage location, Finf model estimates were equal to 0, meaning Finf was equal to 0 and confidence intervals could not be calculated. Although Finf was slightly lower on wildfire days versus non-wildfire days, outdoor generated indoor PM_2.5_ and percentage of indoor PM_2.5_ generated outdoors were both higher on wildfire days compared to non-wildfire days (Table 2).

**Figure 3:**
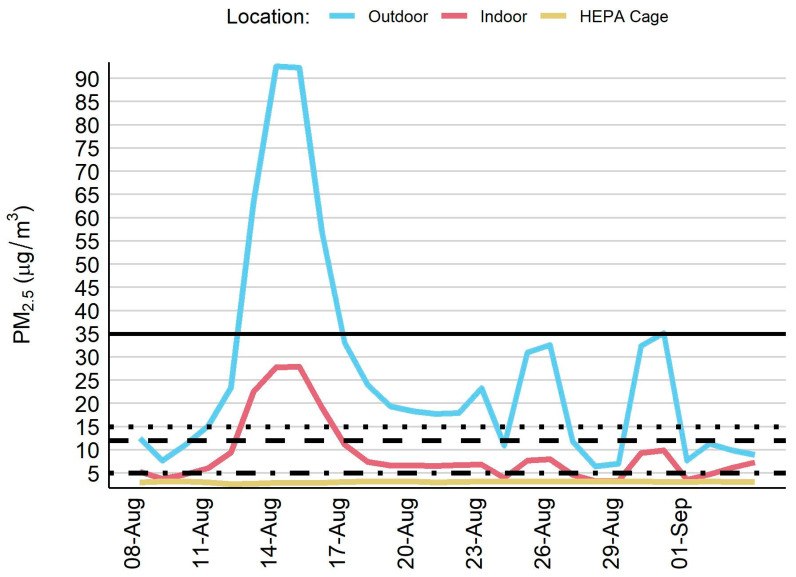
Time series plot for fine particulate matter across three sampling locations. PM_2.5_ = fine particulate matter. Horizontal lines indicate PM_2.5_ thresholds: United State Environmental Protection Agency–24-h standard of 35 µg/m^3^ (solid line) and annual mean of 12 µg/m^3^ (long dashes); World Health Organization–24-h guideline of 15 µg/m^3^ (short dashes) and annual mean of 5 µg/m^3^ (alternating short/long dashes).

During the 28-day sampling period, ambient air quality was negatively impacted and this resulted in exceedances of regulatory thresholds. In particular, there was one remarkable wildfire event which lasted several days in the middle of August where ambient PM_2.5_ concentrations rose above 90 µg/m^3^ (Figure 3). The outdoor PM_2.5_ concentration exceeded the 24-h PM_2.5_ thresholds set by both the WHO (61% of sampling days) and the EPA (18% of sampling days) (Table 3). By contrast, the HEPA cage PM_2.5_ concentration did not exceed the annual or 24-h PM_2.5_ thresholds set by the WHO or the EPA. The indoor room PM_2.5_ concentration values were typically less than the 24-h WHO guideline and EPA standard and the EPA annual standard, except during the major wildfire event that occurred in the middle of August. During this 4-day smoke event, the indoor room PM_2.5_ concentration exceeded all but the EPA 24-h standard.

## 4. Discussion

In this pilot study we demonstrate the potential for ambient air pollution events caused by wildfires to impact indoor air quality within a facility that houses research animals in the western US. To our knowledge, there are no indoor PM_2.5_ standards for public health, occupational health, or research animal health related to PM_2.5_. Thus, we compared our observations to ambient PM_2.5_ public health thresholds provided by the EPA and WHO. Our results show that outdoor PM_2.5_ impacted indoor air quality in the research facility with a remarkable increase in indoor PM_2.5_ during wildfire smoke events. This is notable given that the animal facility and HVAC system in this pilot study are relatively new and utilize the recommended filters designed to capture PM_2.5_.

We hypothesized that infiltration of smoke would be greatest on wildfire days. This would be consistent with others who have identified associations between seasonality and sources of pollution [39]. However, our data revealed that PM infiltration was higher during non-wildfire days within the wildfire season. Interestingly, this is in line with recent data collected by researchers in California who used crowdsourced low-cost sensor data to assess infiltration among residential homes [40]. Liang et al. speculate that infiltration on wildfire days is lower because of behavioral changes like shutting windows and running air conditioning, but it is not clear that these explanations would be relevant in an animal facility. Thus, more research is needed to understand the factors that contribute to changes in infiltration rates. Even though Finf was slightly lower during wildfire days versus non-wildfire days, it is important to reiterate that indoor air quality at the animal facility was adversely impacted by wildfire smoke. Indoor PM_2.5_, outdoor generated indoor PM_2.5_, and percentage of indoor PM_2.5_ generated outdoors were all higher on wildfire days compared to non-wildfire days.

Studies of air pollution toxicology have been conducted in laboratory animals, and this body of evidence informs our presumption that wildfire smoke PM can also cause adverse health effects [41]. However, the biological importance and extent of physiologic effects specific to indoor wildfire smoke exposure remain largely uncharacterized. Without an indoor standard for PM_2.5_, it is difficult to know whether the EPA or WHO ambient thresholds are overly protective or overly relaxed for animals. We speculate this would depend on several factors including animal species, age, and immune status as well as experimental study conditions such as exposure, outcome measure, and duration of study paradigm. The latter would be important especially in circumstances where animals might be episodically and chronically exposed (i.e., exposure to repeated wildfire seasons).

Our finding that wildfire smoke infiltrates animal facilities suggests it is plausible that unintended exposure to smoke could affect the reproducibility of study data. In this way laboratories impacted by smoke might struggle to replicate the findings from laboratories not impacted by smoke, or vice versa. One could argue that for experiments conducted during the wildfire season, “exposed” and “control” animals would both be exposed to the same background level of smoke, and thus any statistical differences may be attributed to the exposure of interest rather than to the wildfire smoke exposure. However, if smoke exposure and the experimental condition of interest acted synergistically to impact an outcome, this larger effect size could be wrongly attributed to the experimental treatment alone. Furthermore, it is particularly problematic to consider studies that use a staggered cohort design where some groups may be raised during fire season while others are not. In these cases, a single lab might struggle to reproduce their own findings from one animal cohort to the next. Smoke exposure could also impact animal breeding operations including breeding success, fertility, and the health of the offspring [19,42,43,44].

Smoke exposure for research animals is a timely and necessary challenge to consider in the US and around the world given that wildfire events continue to increase in frequency and duration concurrent with climate change. Facilities that house animals proximal to prime fire conditions are perhaps at the greatest risk for infiltration-related exposure. However, wildfire smoke is transient and health impacts have been reported in populations living great distances from wildfire events [45]. Some of these distant impacts may be attributed to the differential toxicity reported after “aging” of smoke, which is suggested to produce more oxidative stress [46,47].

Whether smoke exposure occurs locally or downstream of a wildfire event, there is a growing body of data indicating that adverse health outcomes are possible in humans [48] as well as animals [49]. As an example of human effects, a cohort exposed to an intense and long-duration wildfire smoke event in Seeley Lake, Montana, experienced persistent lung function decrements that were measurable two years following exposure [50]. In cell models, wildfire smoke PM has been shown to induce inflammation and cytotoxicity [51]. In guinea pigs, short-term exposure to wildfire smoke can contribute to differential expression of inflammatory cytokines [52]. Effects of wildfire smoke may occur not only in the directly exposed animal but can be passed on to the subsequent generation. In primates, short-term perinatal exposure to wildfire smoke in California resulted in immune modulation that was observable into adolescence in the offspring [53]. Male rats exposed to wildfire smoke produce offspring with behavioral aberrancies, suggesting a potential for multi-generational effects [44]. Such effects could be passed through the germ line as we have demonstrated that prolonged exposure to wildfire smoke significantly alters the sperm epigenome of mice [54]. This and other intergenerational animal studies demonstrate the ability for an exposure to impact the parent generation, the offspring, and even in some cases subsequent generations through inter- and trans-generational inheritance [55]. Such exposure-induced effects within a breeding colony could impact future study outcomes.

With the backdrop of increasing wildfire activity and considering the potential for adverse health outcomes or study confounding, it would be advisable to measure indoor air quality in animal facilities where penetrance of wildfire smoke is possible. From an academic research perspective, the above advisement is consistent with a recent report produced by the University of California Systemwide Air Quality Protocol Working Group which stated “Accurate and reliable outdoor and indoor air quality monitoring and data sources are critical to decision-making related to regulatory compliance, and operational actions” [56]. In the commercial or industrial setting, the EPA suggests using new guidance from the American Society of Heating, Refrigerating, and Air-Conditioning Engineers (ASHRAE) titled “Planning framework for protecting commercial building occupants from smoke during wildfire events” which also suggests that one of the best ways to prepare for wildfire season is to “add the ability to monitor indoor PM_2.5_” [57].

While our specific concern for wildfire smoke is novel and timely, the consideration of air quality more generally in an animal facility and its potential influence on experimental outcomes is not new; Besch reported on this in 1985 [58]. As early as 2003 there were calls for a more thorough description of air quality standards for laboratory animals [59]. Still, there exists no new metric or standard guideline for the measurement or reporting of air quality in laboratory animal facilities in the US. The Canadian Council on Animal Care (CCAC) has provided guidance on indoor air quality including ammonia, carbon dioxide, volatile organic compounds, and PM [60]. The 2019 guidance from the CCAC adopts the EPA’s outdoor annual standard of 12 µg/m^3^ as a maximum threshold for PM_2.5_ in the laboratory animal environment. It is notable that this document does not discuss ambient episodes or sources (e.g., wildfires and smoke events). The lack of acknowledgement for ambient factors by the CCAC and the committee for the Guide for the Care and Use of Laboratory Animals is concerning. It is for this reason that we recommend implementation of air quality monitoring in animal facilities in wildfire-impacted areas to address both indoor and outdoor sources of poor air quality.

The collection of animal facility air quality data would help with decision making within individual facilities and further reporting of this data could inform broader policies and guidelines for laboratory animal environments across the globe. Facilities at risk of ambient exposure to wildfire smoke exposure events should employ active air monitoring programs and develop prudent internal standards and plans for how to deal with aberrant indoor air quality. Building managers, compliance personnel, and research staff should work collaboratively to determine if these air quality disruptions can be predicted and mitigated.

The necessity and scope of air quality mitigation measures will be entirely dependent on each individual facility. Facilities will need to consider the risk for elevated ambient levels of wildfire smoke, anticipated infiltration, and the type of animals or experiments that occur on site. The ASHRAE planning framework outlines several key steps that facilities can take to ensure HVAC systems and buildings are prepared for wildfire season [57]. In this pilot, we demonstrate that a HEPA filter air-supplied mouse rack is sufficient to mitigate exposure under the specific conditions that occurred during the sampling period. More studies will need to be conducted to fully understand the impact that higher levels of infiltration would have on the HEPA filter rack system. However, filter racks may not be available in all facilities and may not be feasible for all species (e.g., large animals). Standalone HEPA filter air purification systems could be an alternative to enhance air quality in large or small animal rooms. Such filters have been shown to substantially reduce indoor air pollution in many settings [61], but the effectiveness in animal research facilities has not been explored. Circumstances including the size of the room, the number of air changes per hour, or the amount of make-up air being brought in from outside the facility could impact the effectiveness of an air purifier. Air quality sensors should be used to attain a baseline and to evaluate any benefits from modifications that are made.

## 5. Conclusions

Wildfire smoke exposure is increasing in certain parts of the US and throughout the world. In this pilot, we demonstrated that PM infiltration occurs in a laboratory animal facility during wildfire season. Universities and other institutions with laboratory animal operations that are at risk of ambient exposure to wildfire smoke should do an indoor air quality inventory, especially during fire season. Whenever possible these institutions should actively monitor the indoor conditions and mitigate infiltration, in order to protect the animals’ health and reduce confounding and loss of confidence in study results.

## Figures and Tables

**Table 1:** Outdoor, indoor, and HEPA cage PM_2.5_ concentrations from 8 August–4 September 2021.

		Outdoor PM_2.5_ (µg/m^3^)	Indoor PM_2.5_ (µg/m^3^)	HEPA Cage PM_2.5_ (µg/m^3^)
	Sampling Days	Mean (sd) Min, Median, Max	Mean (sd) Min, Median, Max	Mean (sd) Min, Median, Max
All Study Days	28	26.2 (23.4) 6.4, 18.1, 92.6	8.9 (6.9) 3.3, 6.7, 27.9	3.1 (0.1) 2.6, 3.1, 3.2
Wildfire Day	12	45.0 (25.4) 23.3, 32.8, 92.6	13.9 (8.1) 7.9, 9.7, 27.9	3.0 (0.2) 2.6, 3.1, 3.2
Non-Wildfire Day	16	12.1 (4.3) 6.4, 11.2, 19.4	5.2 (1.4) 3.3, 5.0, 7.3	3.1 (0.1) 3.0, 3.1, 3.2

PM_2.5_ = fine particulate matter; sd = standard deviation; HEPA = high efficiency purified air; min = minimum; max = maximum. Wildfire Day = day with mean 24-h outdoor PM_2.5_ > 21 µg/m^3^ during wildfire season. Only sampling days with >12 h of hourly data for both indoor and outdoor PM_2.5_ are included in table.

**Table 2:** Comparison of indoor and outdoor air quality data.

	Sampling Days	Outdoor–Indoor PM_2.5_ Difference (µg/m^3^)	Indoor/ Outdoor PM_2.5_ Ratio	Infiltration Efficiency (95% CI)	Outdoor-Generated Indoor PM_2.5_ (µg/m^3^)	Percent (%) Indoor PM_2.5_ Generated Outdoors
		Mean (sd) Min, Median, Max			Mean (sd) Min, Median, Max	Mean (sd) Min, Median, Max
All Study Days						
Indoor location	28	17.3 (16.8) 1.6, 11.4, 64.8	0.34	0.30 (0.21, 0.43)	7.7 (7.0) 1.9, 5.4, 27.8	80 (17) 37, 82, 100
HEPA cage location	28	23.1 (23.5) 3.2, 14.9, 89.7	0.12	NA*	NA*	NA*
Wildfire Day						
Indoor location	12	31.1 (17.6) 14.0, 24.0, 64.8	0.31	0.30 (0.17, 0.50)	13.1 (7.9) 6.8, 9.6, 27.8	94 (8) 74, 100, 100
HEPA cage location	12	42.0 (25.5) 20.1, 29.7, 89.7	0.07	NA*	NA*	NA*
Non-Wildfire Day						
Indoor location	16	6.9 (3.4) 1.6, 6.8, 12.8	0.43	0.40 (0.29, 0.54)	3.6 (1.3) 1.9, 3.4, 5.8	70 (14) 37, 72, 88
HEPA cage location	16	9.0 (4.3) 3.2, 8.0, 16.2	026	NA*	NA*	NA*

PM_2.5_ = fine particulate matter; sd = standard deviation; CI = confidence interval; HEPA = high efficiency purified air; min = minimum; max = maximum. Wildfire Day = day with mean 24-h outdoor PM_2.5_ > 21 µg/m^3^ during wildfire season. Only sampling days with >12 h of hourly data for both indoor and outdoor PM_2.5_ are included in table. NA* = model estimates were equal to 0, meaning infiltration efficiency was equal to 0 and confidence intervals could not be calculated.

**Table 3:** Number of days that sensor measurements surpassed EPA and WHO thresholds.

	Outdoor PM_2.5_ (µg/m^3^)	Indoor PM_2.5_ (µg/m^3^)	HEPA Cage PM_2.5_ (µg/m^3^)
Sampling Days, n	28	28	28
Days with PM_2.5_ > 35 µg/m^3^, n (%) ^a^	5 (18)	0 (0)	0 (0)
Days with PM_2.5_ > 12 µg/m^3^, n (%) ^a^	18 (64)	4 (14)	0 (0)
Days with PM_2.5_ > 15 µg/m^3^, n (%) ^b^	17 (61)	4 (14)	0 (0)
Days with PM_2.5_ > 5 µg/m^3^, n (%) ^b^	28 (100)	20 (71)	0 (0)

PM_2.5_ = fine particulate matter. ^a^ United States Environmental Protection Agency National Ambient Air Quality Standard for PM_2.5_ is 35 µg/m^3^ for a 24-h period and 12 µg/m^3^ for an annual period. ^b^ World Health Organization Air Quality Guideline for PM_2.5_ is 15 µg/m^3^ for a 24-h period and 5 µg/m^3^ for an annual period. Only sampling days with >12 h of hourly data are included in table.

## Data Availability

The data presented in this study are available on request from the corresponding author.
